# Intercostal Lung Herniation

**DOI:** 10.5811/cpcem.2016.12.33212

**Published:** 2017-03-15

**Authors:** David Manthey, Alexander W. Nickle

**Affiliations:** Wake Forest School of Medicine, Department of Emergency Medicine, Winston-Salem, North Carolina

## CASE REPORT

A pleasant 51-year-old gentleman began to experience right chest wall discomfort approximately one month prior to his presentation to the emergency department (ED). He had a history of chronic obstructive pulmonary disease (COPD) and had fractured ribs secondary to coughing paroxysms. He had no history of direct trauma to his chest. He acutely developed a recurrence of his pain and presented to the ED. He stated that he had a coughing paroxysm and developed right-sided posterolateral chest pain. On exam there was a tender, palpable mass in his posterior chest with associated crepitus expanding with inspiration. Portable chest radiograph revealed rib fractures in various stages of healing and right lung outside of his rib cage without pneumothorax. He underwent a computed tomography (CT) of the chest, which confirmed acute rib fractures of ribs 8 and 9 posterolaterally and a chest wall hernia through the eighth intercostal space (Image).

## DISCUSSION

### Intercostal Lung Herniation

Plain radiography revealed a lung herniation outside of the rib cage on the right. A subsequent CT showed acute fractures of the eighth and ninth ribs with herniation of the lung through the eighth intercostal space (Image). Surgery was performed to plate the eighth rib and repair the hernia under thoracoscopy.

Lung herniation is relatively rare and usually occurs in the setting of trauma (penetrating or blunt external force) or surgery.^1^ Spontaneous lung herniation is even more rare, resulting from an increased intrathoracic pressure.^2^ The necessary increase in pressure can be caused by coughing, sneezing, or heavy lifting. The “classic” history for this rare event is sudden onset chest pain after a coughing paroxysm in a male smoker with COPD.^3^ In this case, the patient’s coughing paroxysms were strong enough in force to both fracture ribs and cause a lung herniation. Treatment of lung herniation can include reduction with or without ultrasound guidance, expectant management, and surgical repair.

## Figures and Tables

**Image:**
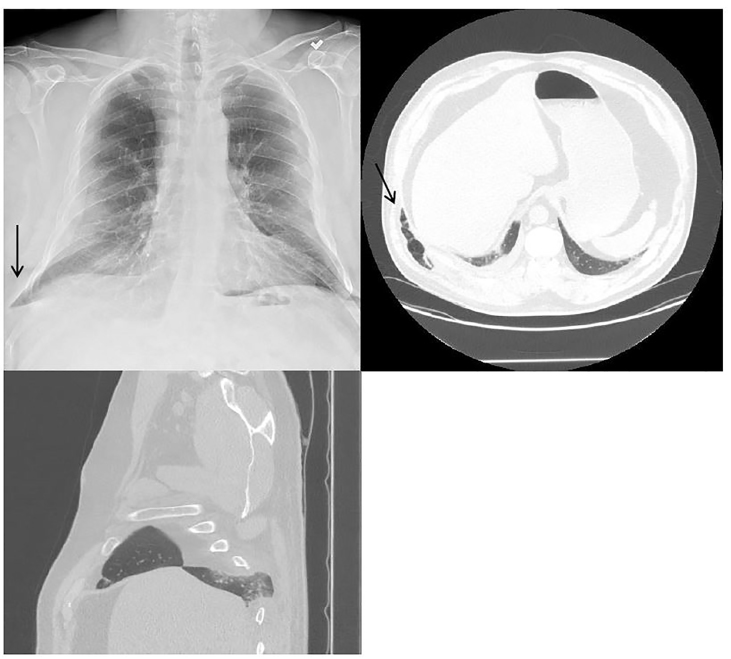
Computed tomography and chest raimgdiograph demonstrating right-sided intercostal lung herniation (arrow).
